# Investigation of water transport in fuel cells using water transport plates and solid plates

**DOI:** 10.1039/c7ra11806f

**Published:** 2018-01-04

**Authors:** Zhiqiang Wang, Lijuan Qu, Yachao Zeng, Xiaoqian Guo, Zhigang Shao, Baolian Yi

**Affiliations:** Fuel Cell System and Engineering Group, Dalian Institute of Chemical Physics, Chinese Academy of Sciences 457 Zhongshan Road 116023 Dalian PR China zhgshao@dicp.ac.cn +86 411 84379185 +86 411 84379153; University of Chinese Academy of Sciences 19A Yuquan Road 100049 Beijing PR China

## Abstract

Water management of proton exchange membrane fuel cells (PEMFCs) is of vital importance to achieve better performance and durability. In this study, porous hydrophilic water transport plates (WTPs) with different pore structures were prepared and employed to improve water management in PEMFCs. Polarization curves, electrochemical impedance spectroscopy (EIS) and water balance were tested to investigate the effect of pore structure on cell performance and water transport process. The results show that pore structure has little effect on drainage function due to excess liquid water flux of WTPs, while the membrane hydration is improved with increased surface evaporation rate of WTPs, resulting in better cell performance. The favorable cell performance shows that WTP is a promising technique to improve water management in PEMFCs.

## Abstract 2

Water management of proton exchange membrane fuel cells (PEMFCs) is of vital importance to achieve better performance and durability.

## Graphical abstract

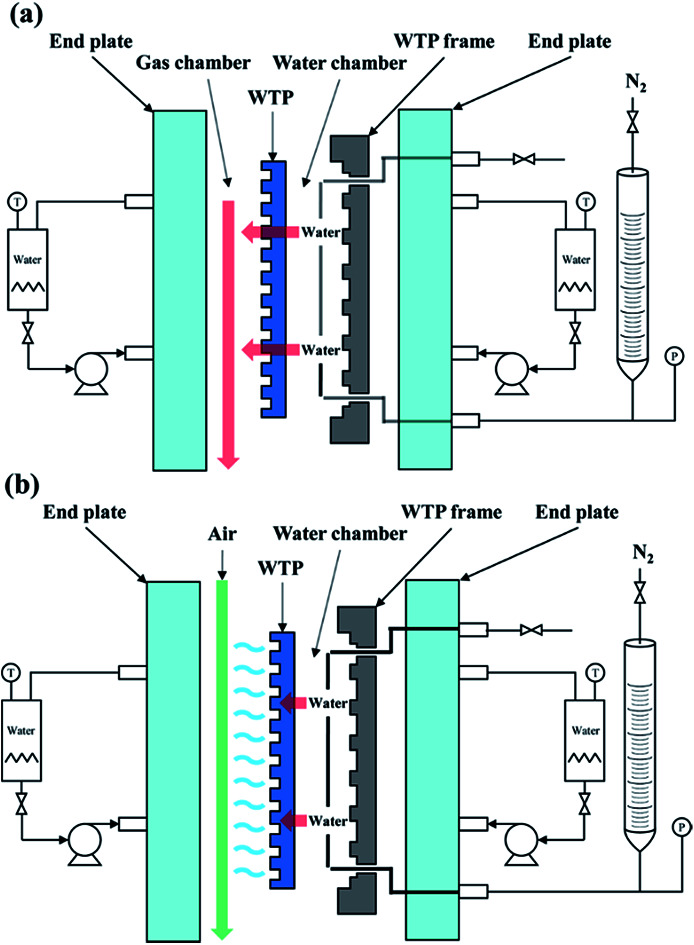

## 1. Introduction

Proton exchange membrane fuel cells (PEMFCs) have attracted much interest due to their potential applications in portable power, vehicle power supplies and power stations.^1–3^ One of the critical issues associated with performance and durability is water management.^4,5^ Perfluorosulfonic acid (PFSA) membranes (*e.g.*, Nafion membrane) have been widely used in a commercial PEMFC, the water content of the PFSA membrane has a significant influence on cell performance and durability. Sufficient water is necessary for the membrane to obtain enough ionic conductivity. Meanwhile, excess liquid water should be timely removed in case of flooding in porous media such as the gas diffusion layer (GDL) and catalyst layer (CL).^6–8^ The balance between membrane dehydration and electrode flooding must be guaranteed in PEMFCs.

Various approaches have been proposed to improve water management in PEMFCs, including external humidifier,^9,10^ internal humidifier,^11,12^ self-humidification,^13–15^ dynamic drainage^16,17^ and so on. Another promising technique is water transport plate (WTP), which is firstly proposed by AT 389020 (ref. 18). The WTP in the patent is a porous plate serving as bipolar plate and WTP can humidify inlet gas without external humidifier. Afterwards, United Technologies Corporation Power (UTC Power) improved WTP technology, which can not only humidify inlet gas streams to ensure enough ionic conductivity of membrane but also remove liquid water to avoid electrode flooding.^19–22^ Compared to other water management strategies, WTPs can simultaneously balance membrane dehydration and water flooding. The Puremotion Model 120 Fuel Cell Power System with WTPs has been applied in vehicles and achieved over 25 000 h of operation without a fuel cell failure, which enlightens the great potential of WTP technology to improve performance and durability of PEMFCs.^23,24^

Shawn *et al.*^25^ compared cell performance of traditional solid plate (SP) and porous carbon plate under dry feeds condition. The performance and stability of fuel cell were significantly improved after applying porous plate, especially under lower stoichiometry. Wang *et al.*^26,27^ studied water and heat transfer through WTP, which aimed at improving water and heat management in fuel cell stacks. Guo *et al.*^28,29^ investigated the effects of the WTPs properties on the cell performance and stability. Weber *et al.*^22^ developed a one dimensional model to simulate the fuel cells with WTPs and SPs, the differences of water distribution were discussed. Previous research showed the great advantages of WTPs, but water transport in fuel cell with WTP was not fully understood.

In this study, WTPs with different pore structure were prepared and employed in PEMFCs. Water transport process was investigated by water balance tests. Furthermore, the effects of WTPs' pore structure on cell performance and water transport were discussed in detail.

## 2. Experimental

### 2.1. Preparation of WTPs and SP

In order to investigate effects of pore structure on water transport in the fuel cell, WTPs with three different pore structure, which were marked as WTP-1, WTP-2 and WTP-3, were prepared while the SP served as baseline. First, the WTPs and SP (Bought from Shanghai Hongfeng Co, Ltd.) were machined into required shape by computer numerical control (CNC) machine. To improve the hydrophilicity of porous plates, WTPs were treated by impregnation method.^30^ The WTPs were vacuum impregnated in the titanium dioxide (TiO_2_) sol solution prepared by sol–gel method until no bubble was observed in solution. Then the samples were taken out and heated in the air at 400 °C for 2 h.

### 2.2. Characterization of WTPs and SP

To investigate the effect of pore structure on physical properties, various physical characterizations of WTPs and SP were tested. Scanning electron microscopy (SEM, JSM-IT300) was conducted to investigate the pore structure of the samples. The pore size, porosity and tortuosity of the WTPs were measured by mercury intrusion porosimetry (PoreMasterGT 60). The in-plane electronic conductivity was measured by a four-point probe detector (Suzhou Jingge Electronic Co, Ltd. ST-2258A). The interfacial contact resistance (ICR) was tested by a universal testing machine (Changchun Kexin Test Instrument Co, Ltd. WDW-1010) at a clamping pressure of 1.5 MPa. To characterize the wettability between samples and water, the contact angle test was conducted on a drop shape analyzer (KRUSS, DSA 100) by a sessile drop method. The three-point bending test was carried out by the same universal testing machine according to GB/T 13465.2-2014.^31^ The flexure strength can be calculated by equation eqn (1).1
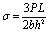
where *σ* is the flexure strength of samples (MPa); *P* is the breaking force (N); *L* is the support span distance (50 mm); *b* is the width of samples (10 mm); and *h* is the thickness of the samples (3 mm).

**Fig. 1:**
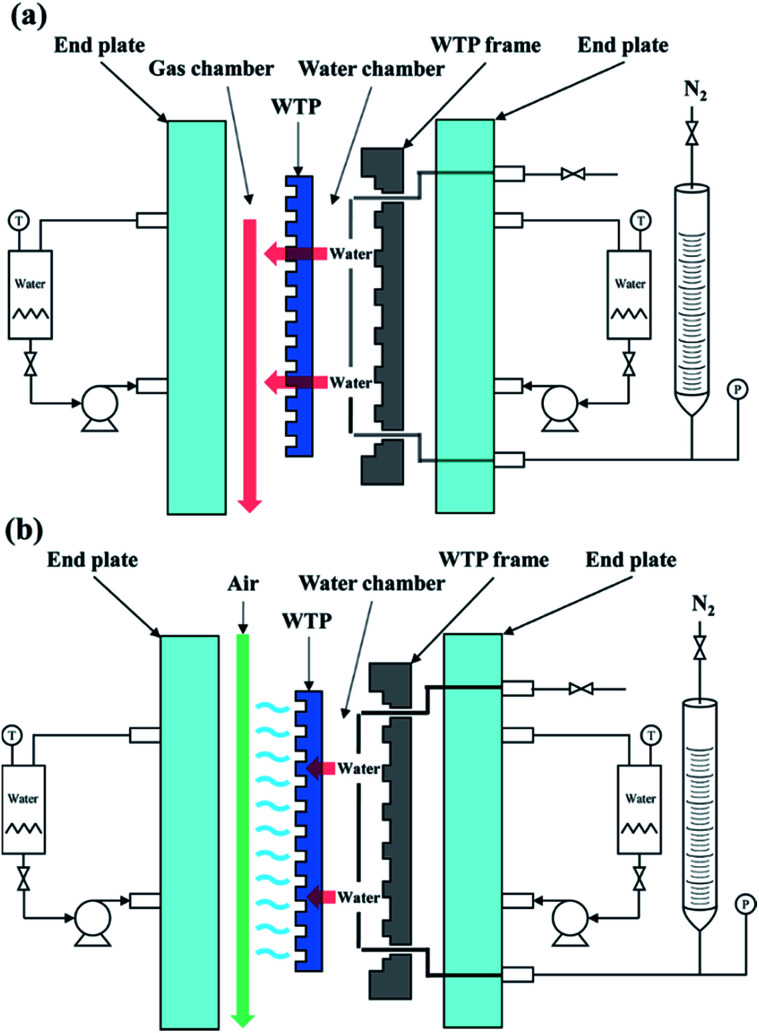
Schematic diagram of (a) liquid water flux test apparatus and (b) surface evaporation rate test apparatus.

In addition, WTPs should separate hydrogen and oxygen while transfer water at the same time. Bubble pressure measurement was carried out on a home-made apparatus^29^ to investigate gas-blocking property of WTPs. To ex-situ characterize the humidification and drainage capability of WTPs, liquid water flux and vapor water surface evaporation rate were measured by a test apparatus similar to a fuel cell, as shown in Fig. 1. The test parameters simulate the operating conditions of PEMFC. The WTPs were machined with flow field and sealed into the apparatus with an efficient area of 50 cm^2^. The temperature of the system was kept at 60 °C by circulating water. When liquid water flux was evaluated, the water chamber was given a certain pressure while the gas chamber was exposed to the ambient air. The water can permeate through WTP. The liquid level change of pipette connecting with water chamber could be recorded and the liquid water flux could be calculated. When surface evaporation rate was measured, the absolute pressure of water chamber was 0.13 MPa while the gas chamber was 0.15 MPa. The flow rate of dry air in gas chamber was fixed. The water can evaporate on the surface of WTP to humidify unsaturated gas. The calculation method of surface evaporation rate was similar to the one of liquid water flux.

### 2.3. Single cell test

Single fuel cell test with SP and WTPs were conducted to investigate the effects of pore structure on cell performance and water transport process. Catalyst coated membrane (CCM) was prepared by spraying Pt/C (70 wt%, Johnson Matthey) onto Nafion® 211 membrane (Dupont) with Pt loading of 0.2 mg cm^−2^ at anode and 0.4 mg cm^−2^ at cathode. Afterwards, membrane electrode assembly (MEA) with 50 cm^2^ active area was prepared by sandwiching the CCM between two gas diffusion layers (GDLs). A special single cell was designed to evaluate the humidification and drainage function of WTPs.^32^ Parallel gas flow field was machined on both anode and cathode side with the following dimensions: channel width of 1 mm, rib width of 1 mm and channel depth of 0.4 mm. The gas flow rates were switched with fixed stoichiometry, which is 2.0 for hydrogen and 3.0 for air. The absolute pressure of the gas chamber was 0.15 MPa while that of coolant chamber was 0.13 MPa. Cell temperature was 60 °C. The operating conditions were outlined if there is no special description.

**Fig. 2:**
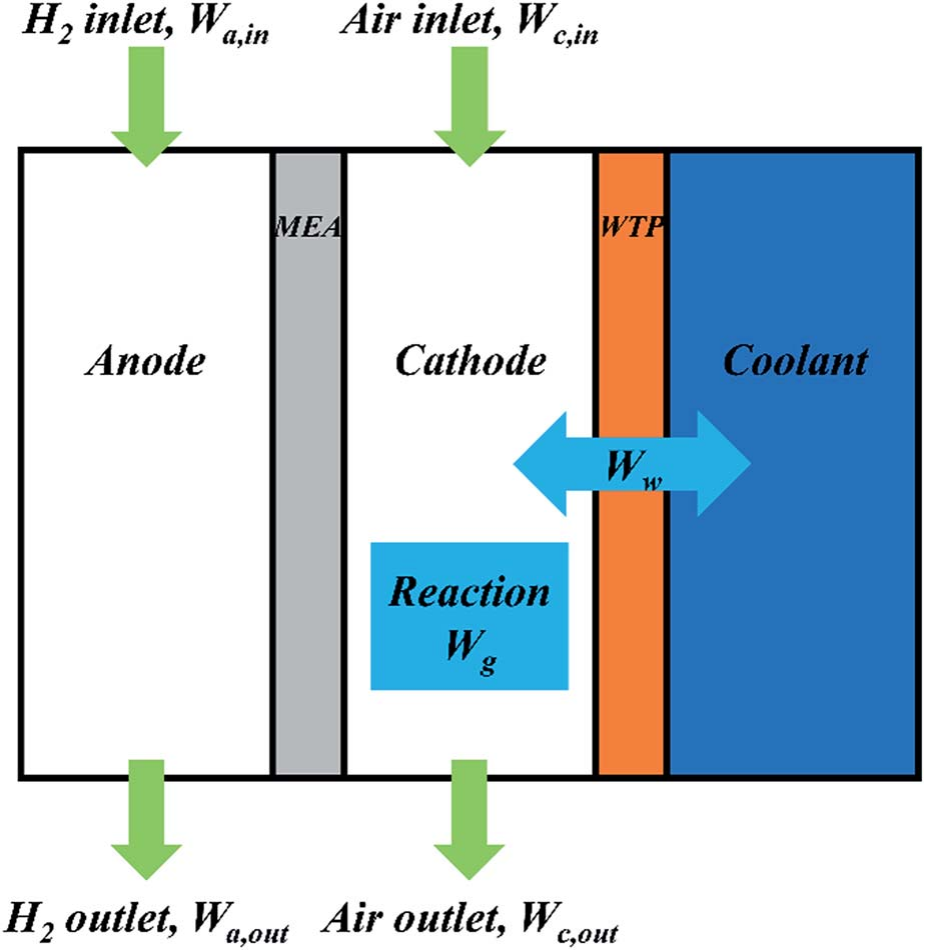
Schematic diagram of water transport process in PEMFC.

Fuel Cell Test System (Scribner, 850E) was used for evaluating single fuel cell performance. All the polarization curves were recorded after 8 h's activation at 1000 mA cm^−2^. The data was recorded every 50 mA cm^−2^ and each point was remained for 30 seconds. The fuel cell was conditioned at 1000 mA cm^−2^ for at least 30 minutes to reach a steady state before the measurement of electrochemical impedance spectroscopy (EIS). Then EIS was measured with a frequency range of 10 kHz to 0.1 Hz and 10% amplitude of current. Cyclic voltammogram was taken to obtain the electrochemical surface areas (ECSAs) of cathode catalyst layer. The hydrogen and nitrogen were provided at flow rates of 0.3 L min^−1^. The potential was swept from 0.08 V to 1.20 V (*vs.* RHE) at a scan rate of 50 mV s^−1^. Afterwards, ECSAs were calculated from the areas of hydrogen desorption peak. In order to *in situ* study water transport inside fuel cell, water balance was tested. The fuel cell was conditioned at 1000 mA cm^−2^ to reach a steady state before the test. Desiccator was added respectively at anode outlet and cathode outlet to collect water in gas chamber. A pipette was used to display water change in water chamber. Fig. 2 shows schematic diagram of water transport process in a PEMFC. According to the conservation of mass, the following equation should be established.

2 *W*_a,in_ + *W*_c,in_ + *W*_g_ = *W*_a,out_ + *W*_c,out_ + *W*_w_

where *W*_a,in_ and *W*_c,in_ are water content of anode and cathode inlet; *W*_a,out_ and *W*_c,out_ are water content of anode and cathode outlet; *W*_w_ is water flux through WTP and positive flux means that water transfers from gas chamber to coolant chamber. The above values can be obtained by measurement whereas generated water, namely *W*_g_, can be calculated by eqn (3).3
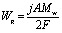
where *j* is current density; *A* is active area of the cell; *M*_w_ is molecular weight of water; *F* is Faraday constant.

In addition, the water input amount, *W*_in_, the water output amount, *W*_out_ and experimental error can be defined by the following equations.

4 *W*_in_ = *W*_a,in_ + *W*_c,in_

5 *W*_out_ = *W*_a,out_ + *W*_c,out_ + *W*_w_

6
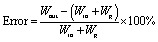


## 3. Results and discussion

### 3.1. Characterization of SP and WTPs

#### 3.1.1. Microstructure characterization

**Fig. 3:**
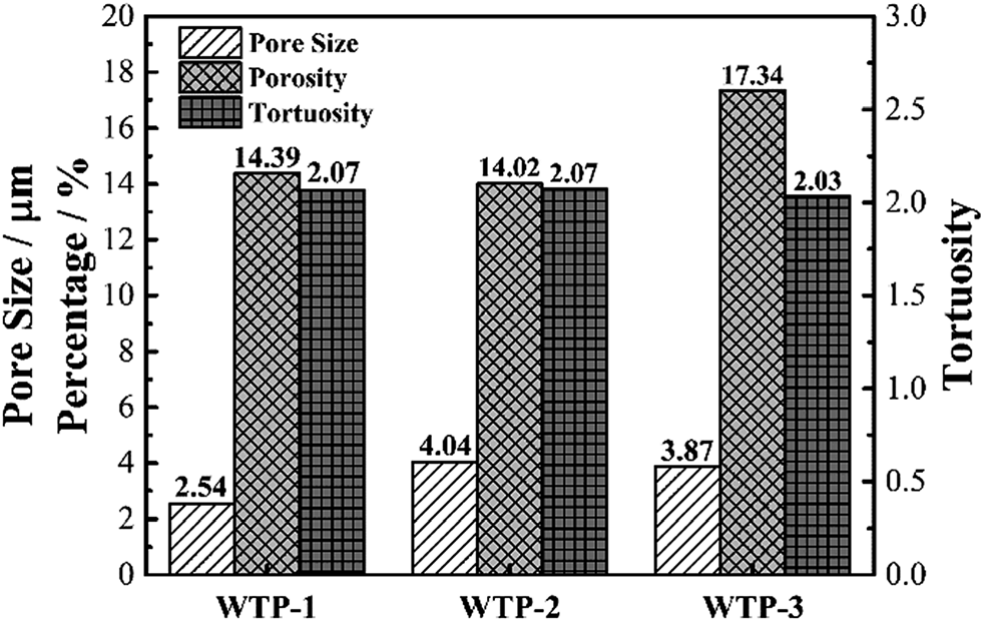
Mean pore size, porosity and tortuosity of water transport plates with different pore structure.

**Fig. 4:**
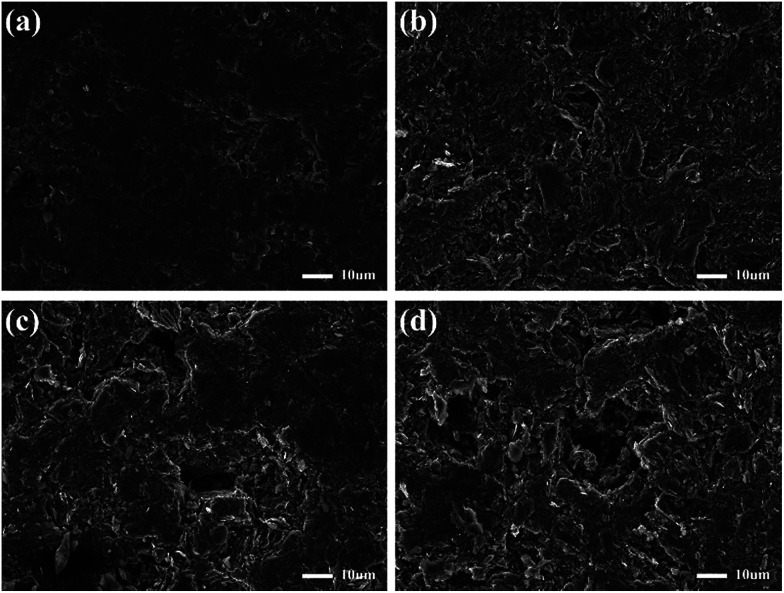
SEM images of the solid plate and water transports plates with different pore structure ((a) SP, (b) WTP-1, (c) WTP-2, (d) WTP-4).

The pore structure parameters of the samples were shown in Fig. 3. Since the graphite powder used to prepare the graphite plate has different particle size, the WTPs have different pore size and porosity while similar tortuosity, resulting in different water permeability. To further confirm the pore structures of samples, SEM images were shown in Fig. 4. As can be seen, there is almost no crevice on the surface of SP and the surface roughness is the lowest of the samples. With increased particle size of graphite powder, the pores and surface roughness on the surface of the samples are continuously increasing.

#### 3.1.2. Electronic conductivity measurement

**Fig. 5:**
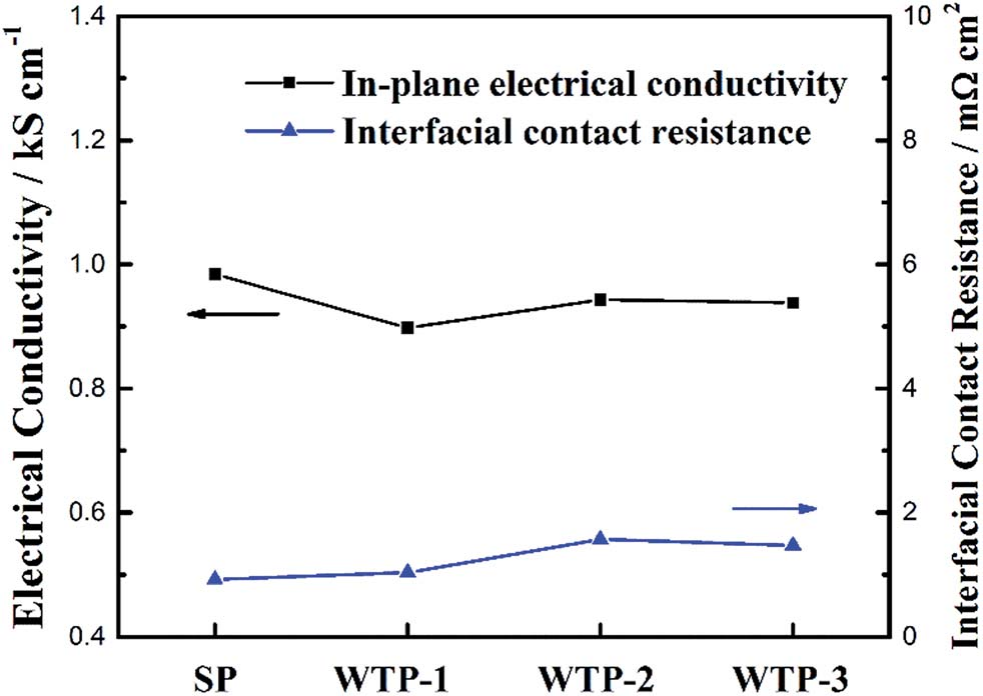
The in-plane electrical conductivity and ICR of SP and WTPs.

The in-plane electrical conductivity and ICR of SP and WTPs were shown in Fig. 5. As can be seen, WTPs have slightly poor electrical conductivity compared with SP. While WTPs with different pore structure have similar in-plane electrical conductivity and ICR, which means pore structure has little impact on electronic conductivity. Thus, the influence of electronic conductivity on cell performance can be ignored.

#### 3.1.3. Mechanical property characterization

**Fig. 6:**
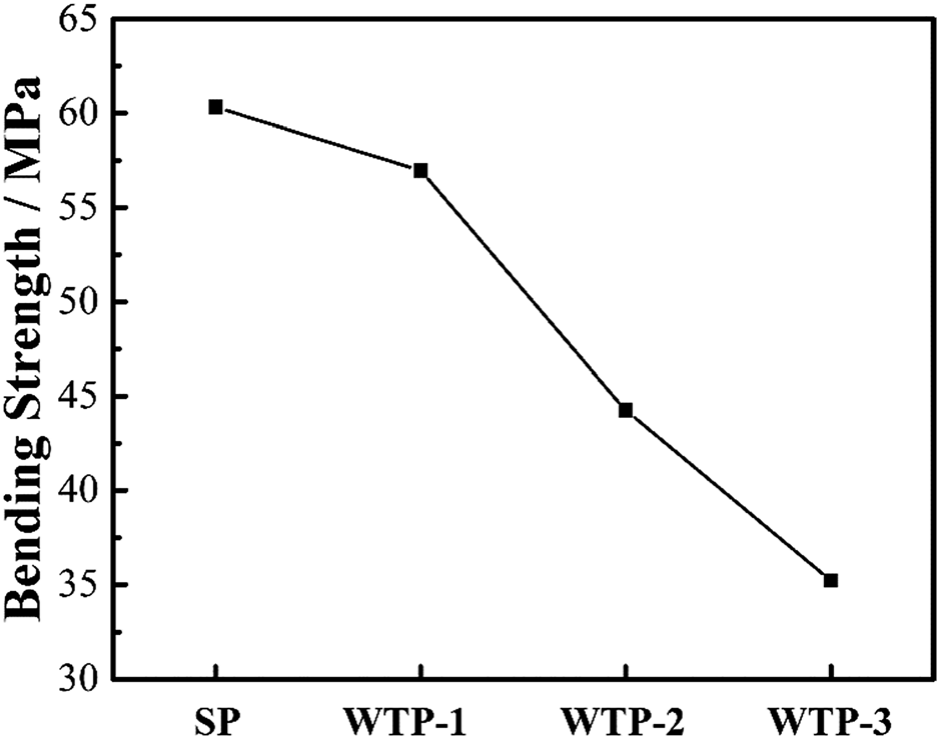
Bending strength of SP and WTPs.

The flexure strength data was shown in Fig. 6. It can be seen that SP has the best mechanical property while the bending strength reduces gradually. This is mainly attributed to the presence of pore structure. In Fig. 4, the samples have continuously increasing pores, resulting in reduced bending strength.

#### 3.1.4. Wettability characterization

**Fig. 7:**
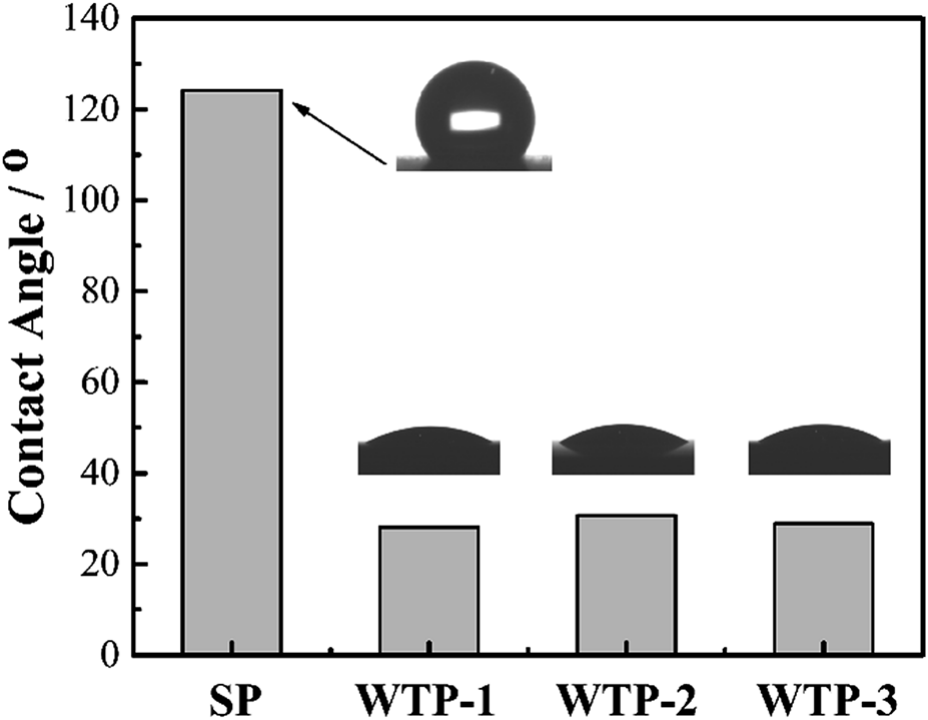
Contact angles of SP and WTPs.

The contact angle of SP and WTPs were shown in Fig. 7. Compared with traditional SP, WTPs show good hydrophilicity after hydrophilic treatment. However, the pore structure has little effect on wettability of WTPs.

#### 3.1.5. Bubble pressure measurement

The data of bubble pressure of samples were listed in Table 1. The bubble pressure of WTP reduces gradually.

**Table 1:** Bubble pressure of the samples

Samples	Bubble pressure/MPa
SP	—
WTP-1	0.17
WTP-2	0.11
WTP-3	0.08

According to Young's equation, the capillary pressure of WTP is inversely proportional to the characteristic pore size, which means bubble pressure decreased with an increase in maximum pore size. However, the actual bubble pressure of WTPs in fuel cell is lower than the measured value. This is mainly due to flow fields and higher temperature. In single cell tests, WTP-3 could lead to gas crossover when the pressure differential between cathode chamber and coolant chamber is 0.04 MPa. The poor gas-blocking capability of WTP-3 will lower the reliability and stability of fuel cell, which is not recommended in real application.

#### 3.1.6. Liquid water flux measurement

**Fig. 8:**
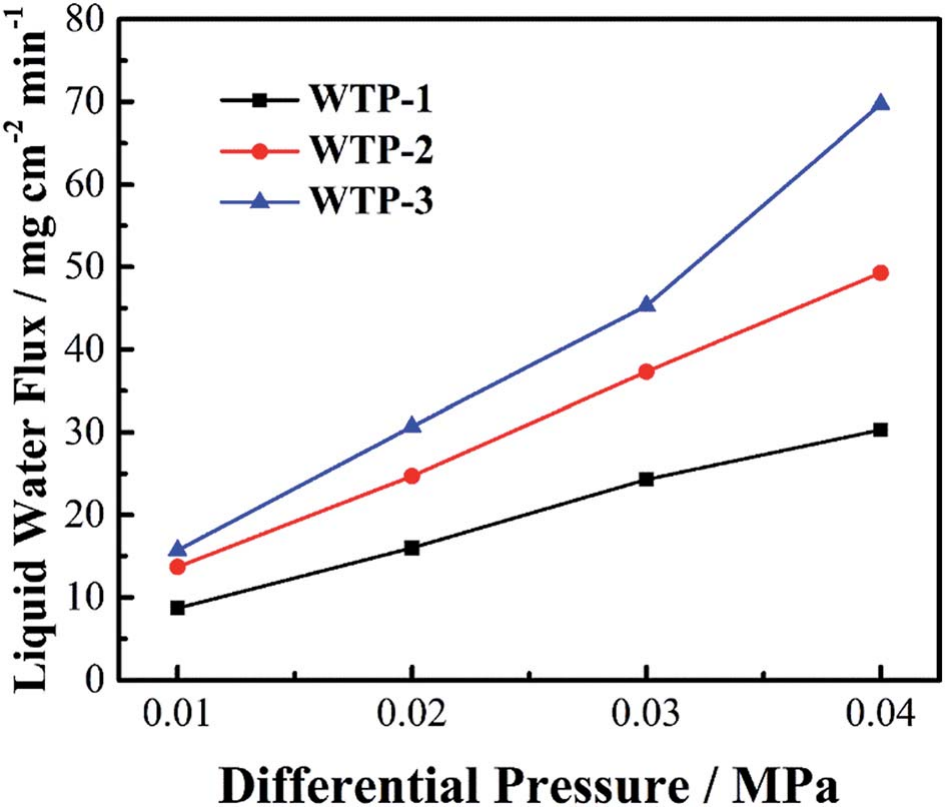
Liquid water flux of the samples.

As shown in Fig. 8, the liquid water flux has a linear relationship with differential pressure between gas chamber and water chamber. Meanwhile, the water flux changes with different pore structure.

The water permeability process is determined by Darcy's law:7
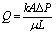
where *Q* is the water flux, *k* is the water permeability, *A* is the area of the samples, Δ*P* is the differential pressure between two chamber, *μ* is the viscosity of water, *L* is the thickness of the samples.

According to eqn (7), the liquid water flux is proportional to *k* and Δ*P*. The water permeability, *k*, is determined by pore structure of the samples. Generally, the water permeability increases with bigger pore size and porosity.^28^ Thus, the pore structure has a great influence on water permeability, resulting in different liquid water flux.

#### 3.1.7. Surface evaporation rate measurement

**Fig. 9:**
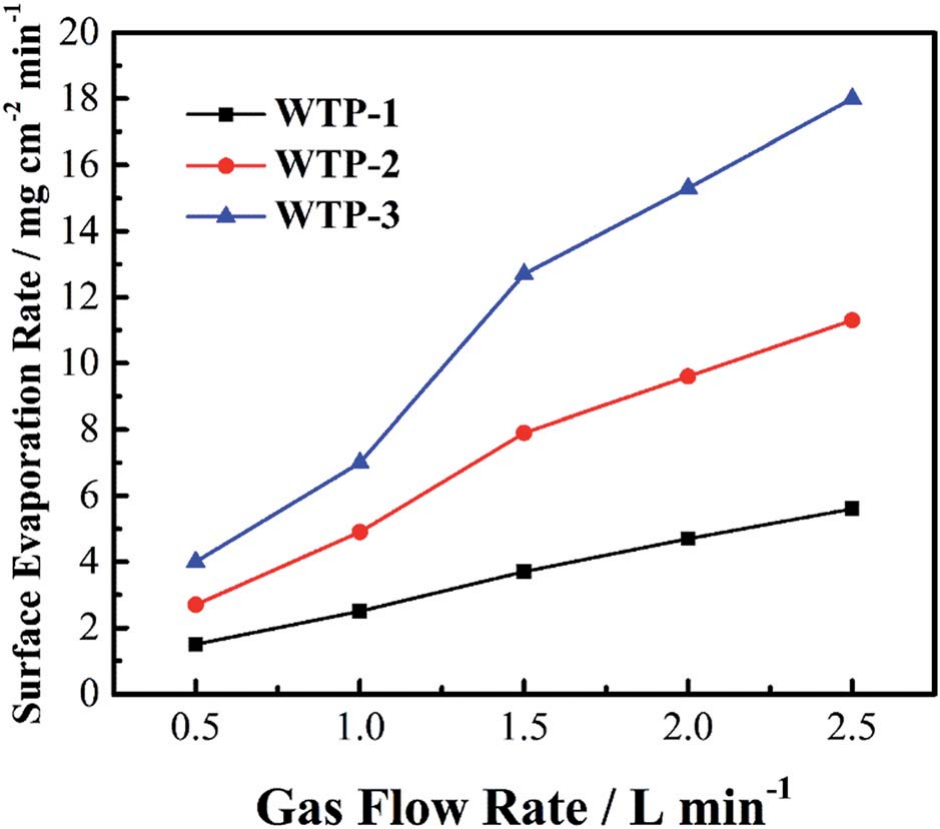
Surface evaporation rate of the samples.

The surface evaporation rate of the samples were shown in Fig. 9. The evaporation rate increased with increasing gas flow rate. Meanwhile, the surface evaporation rate changes with different pore structure.

As for the evaporation process, the following equation can be used to describe the water evaporation rate:^22^8
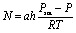
where *N* is the evaporation rate, *a* is the ratio of wetted area to active area, *h* is the evaporation coefficient of vapor water, *P*_sat_ is the saturated vapor pressure of water, *P* is the water partial pressure in gas chamber, *R* is the universal gas constant, and *T* is the temperature. According to eqn (8), the evaporation rate is proportional to *a* and *h*. The parameter *a* is related to pore size and porosity while the coefficient *k* has a linear relationship with gas flow rate.^33^

In summary, pore structure has little effect on electronic conductivity and wettability but has a great influence on flexure strength, bubble pressure and water flux. Since the water flux is a key parameter for the humidification and drainage process of WTP,^29^ the following discussion takes the liquid water flux and surface evaporation rate as the independent variable.

### 3.2. Effect of liquid water flux on drainage function of WTPs

**Fig. 10:**
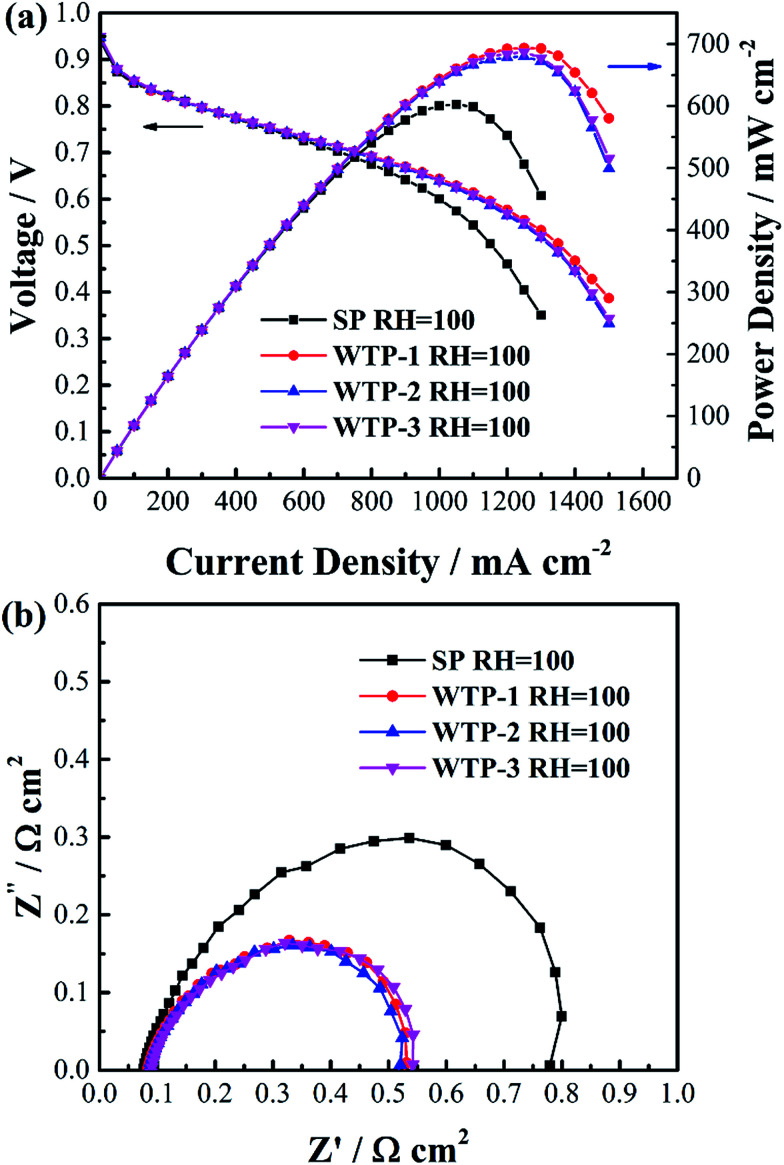
(a) Polarization curves and (b) electrochemical impedance spectroscopy at 1000 mA cm^−2^ of SP and WTP under saturated humidified condition.

To investigate the influence of liquid water flux on drainage function of WTPs, cell performance of SP and WTPs with different water flux were evaluated respectively. The inlet gas streams were saturated humidified to eliminate the effect of humidification process. WTP was placed at cathode side. The polarization curves are shown in Fig. 10(a). The cell performance is significantly improved after replacing SP with WTP. The enhanced performance is due to drainage function of WTPs. However, WTPs with different pore structure show similar cell performance.

To further study the reason, EIS was tested, as seen in Fig. 10(b). The main difference between SP and WTP is the radius of low frequency arc. This can be attributed to a weakened flooding issue due to WTP's drainage function, resulting in reduced mass transport loss. Meanwhile, the water balance test in Table 2 can obtain the same inference. Water content of cathode chamber is reduced sharply after applying WTP, which can mitigate flooding phenomenon in cathode GDL and CL. Moreover, the water content in the anode chamber increased after employing WTP, which is probably due to temperature-driven water transport. Meanwhile, the EIS and water balance test with different liquid water flux show relatively constant results. Theoretically, increased liquid water flux of WTPs means better drainage function. The main reason is the surplus liquid water flux. The generated water flux is 5.60 mg cm^−2^ min^−1^ at 1000 mA cm^−2^. While the minimum liquid water flux is 16.0 mg cm^−2^ min^−1^, which is still higher than the generated water. In saturated humidified condition, the quantity of excess water is basically equal to the generated water, which should be removed. As seen in Table 2, almost all of the generated water can be removed by WTPs and the remaining water is blown out by exhaust. Both of the three WTPs are capable of mitigating electrode flooding due to the surplus drainage function. As a result, the WTPs show similar drainage function in fuel cell operation.

**Table 2:** Water balance test of SP and WTP under saturated humidified condition

Samples	Water flux/mg cm^−2^ min^−1^								Error%
	*W*_a,in_	*W*_a,out_	*W*_c,in_	*W*_g_	*W*_c,out_	*W*_w_	*W*_in_ + *W*_g_	*W*_out_	
SP	2.00	1.98	4.20	5.60	9.89	—	11.80	11.87	0.56
WTP-1	2.00	2.31	4.20	5.60	4.79	5.07	11.80	12.16	3.05
WTP-2	2.00	2.15	4.20	5.60	4.61	5.20	11.80	11.96	1.36
WTP-3	2.00	2.20	4.20	5.60	4.32	5.20	11.80	11.72	−0.68

### 3.3. Effect of surface evaporation rate on humidification function of WTPs

**Fig. 11:**
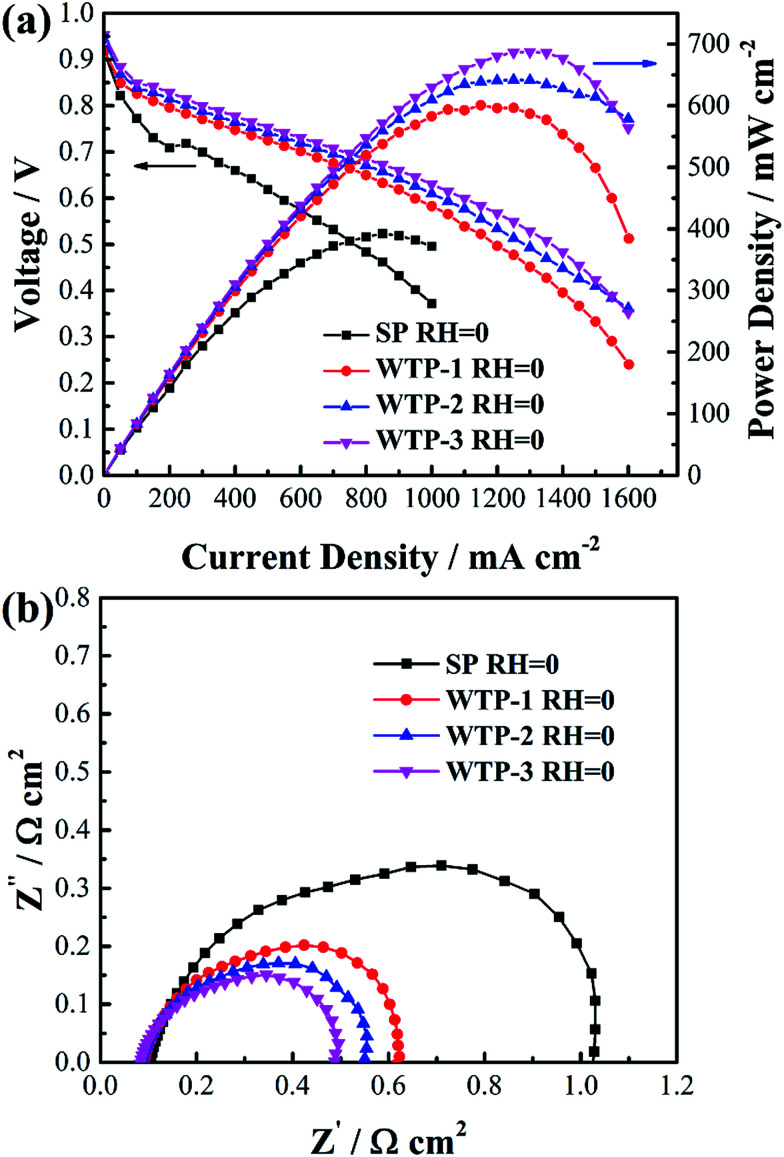
(a) Polarization curves and (b) electrochemical impedance spectroscopy at 1000 mA cm^−2^ of SP and WTP fuel cell under zero inlet humidity.

In order to investigate effect of surface evaporation rate on humidification function of WTPs, cell performance of SP and WTPs with different surface evaporation rate were evaluated respectively. The inlet gas humidity was zero and WTP was placed at cathode side. The polarization curves are shown in Fig. 11(a). Compared with traditional SP, cells with WTP installed show better performance. And cell performance increased with larger surface evaporation rate. Moreover, benefiting from humidification and drainage function, WTP type fuel cell without external humidification showed comparable performance to SP type fuel cell with saturated humidity feeds, which can lighten and simplify the whole system.

To explain enhanced cell performance, EIS was measured at 1000 mA cm^−2^, as shown in Fig. 11(b). It can be seen high frequency resistance (HFR) of cell with WTP is smaller than the one with SP, and HFR decrease with increased surface evaporation rate. The main reason is reduced membrane resistance due to better membrane hydration. The water can transfer from water chamber to gas chamber through WTP. The larger the surface evaporation rate, the smaller the HFR. Another difference is the radius of low-frequency arc, which is composed of electrochemical polarization and mass transfer polarization. To explain this, ECSAs were measured as shown in Table 3. The increased ECSA reveals higher catalyst utilization due to a better membrane hydration, which can lower electrochemical polarization. Resulting from more electrochemical active sites, the diffusion length of oxygen is shorter, which reduces the mass transport polarization.^34^

**Table 3:** ECSA of fuel cells with SP and WTPs under zero humidity condition

Samples	RH	ECSA/m^2^ g_Pt_^−1^
SP	0	20.10
WTP-1	0	53.49
WTP-2	0	53.74
WTP-3	0	56.34
SP	100%	66.33

For further study water transport process inside fuel cell, water transport was tested as shown in Table 4. The smallest anode water content is mainly due to lack of back diffusion at inlet of fuel cell resulting from lowest gas humidity. And the reason for high cathode water content is that water in cathode is all blown out by dynamic drainage. The addition of WTP at cathode side will humidify the air in cathode and enhance back diffusion. Thus, an increase in anode water content is observed. With increased water flux, water content in cathode increases and water content in water chamber decreases. Moreover, the net water transport direction changes. As we mentioned above, the static drainage capacity of different WTP is basically same under fuel cell operation. Thus, the main reason should be their different humidification capacity, which is consistent with the above analysis.

**Table 4:** Water balance of fuel cells with SP and WTP at 1000 mA cm^−2^ under zero humidity condition

Samples	Water flux/mg cm^−2^ min^−1^								Error%
	*W*_a,in_	*W*_a,out_	*W*_c,in_	*W*_g_	*W*_c,out_	*W*_w_	*W*_in_ + *W*_g_	*W*_out_	
SP	0	0.91	0	5.60	4.79	—	5.60	5.70	1.79
WTP-1	0	1.51	0	5.60	4.09	0.13	5.60	5.73	2.26
WTP-2	0	1.41	0	5.60	4.37	0.07	5.60	5.85	4.40
WTP-3	0	1.36	0	5.60	5.01	−0.80	5.60	5.57	−0.60

## 4. Conclusion

Porous graphite plates, serving as water transport plates, were employed in fuel cell to improve water management. WTPs with different pore structure have similar electronic conductivity and wettability. Whereas the flexure strength and bubble pressure decrease, while the liquid water flux and surface evaporation rate increase. Beneficial from the humidification and drainage function of WTPs, cell performance was successfully improved under saturated or zero humidified feeds conditions. The WTPs with different liquid water flux have similar drainage capability due to excess liquid water flux. Moreover, the cell performance can be promoted with an increase in surface evaporation rate. Taking into account of mechanical property, gas-blocking property and cell performance, WTP-2 is a favorable choice to improve water management in fuel cells. The enhanced performance indicates that water transport plate is a promising technique in PEMFCs.

## Conflicts of interest

There are no conflicts to declare.

## Supplementary Material
